# An exploration of Michigan certified peer support specialists’ perceptions on tobacco use and additional future supports

**DOI:** 10.18332/tpc/200025

**Published:** 2025-02-24

**Authors:** Sahana Lothumalla, Devin C. Tomlinson, Isabelle Duguid, Chelsea Wilkins, Natalie D. Bayrakdarian, Lauren Hellman, Mary Jannausch, Pam Werner, Adrienne Lapidos, Lara N. Coughlin

**Affiliations:** 1 Addiction Center, Department of Psychiatry, University of Michigan, Ann Arbor, United States; 2 Bureau of Community Based Services, Lansing, United States; 3 Program for Mental Health Innovation, Services, and Outcomes, University of Michigan Medical School, Ann Arbor, United States

**Keywords:** peers, tobacco cessation, tobacco, rurality

## Abstract

**INTRODUCTION:**

Certified peer support specialists, recovery coaches and community health workers uniquely connect to individuals who smoke through shared experiences. This study examines peers' perceptions of tobacco cessation supports to enhance policy and intervention opportunities in rural and non-rural communities.

**METHODS:**

Peers (n=172) responded to a needs assessment available from 30 November 2023 to 1 February 2024. Peers were asked questions about their perceptions of currently available and additional support for tobacco cessation. We report overall ratings of these services, as well as ratings stratified by rurality.

**RESULTS:**

Over half of peers perceived widely available tobacco cessation services (Quitline, counseling, NRT, medications, peer-to-peer support) as somewhat effective. Peers tended to favor financial accessibility, holistic health approaches, flexible approaches focused on harm reduction, and increased tobacco cessation services awareness, as well as peer support as additional services. When stratified by rurality, more rural-residing peers reported current tobacco cessation services as at least somewhat effective, but called for greater access to these services and/or the need for novel approaches specifically for individuals in rural communities. Rural peers emphasized the importance of more holistic support, cessation services awareness, virtual opportunities, peer-led services, and healthcare provider education for stigma prevention than their urban counterparts.

**CONCLUSIONS:**

Most peers view existing supports as somewhat effective, with peer-to-peer support rated highest. Rural-residing peers favored holistic and virtual supports and urban-residing peers emphasized harm reduction and healthcare coverage, suggesting future cessation efforts within the peer workforce should address rural-specific barriers and leverage community-centered, flexible approaches.

## INTRODUCTION

Tobacco use is the leading preventable cause of disease and death in the United States, contributing to over 480000 deaths each year^1^. Tobacco use causes detrimental health effects, including cancer and heart disease^2^. Cigarette smoking is linked to 80–90% of lung cancer diagnoses, corresponding to a 5–10 times increase in lung cancer risk for those who smoke^3,4^.

The health risks of cigarette smoking are widely acknowledged; however, quitting remains challenging, with only 8% of quit attempts resulting in cessation^5^. The chemicals in tobacco, particularly nicotine, can lead to physical dependence and cause withdrawal symptoms^6^. Tobacco dependence is unique to the individual, with genetic, physical, and social factors influencing an individual’s pathway to cessation^7^. Services including nicotine replacement therapy (NRT), tobacco cessation counseling, Quitline services, and prescription medications are commonly used to aid tobacco cessation. NRT supplies nicotine in alternative forms to relieve physical withdrawal symptoms^8^. Quitlines offer coaching and program referrals^9^. Prescription medications can help lower withdrawal symptoms^10^. In Michigan, the location of the present study, 15.2% of adults smoke and over 16000 adults die each year from smoking-related deaths, showcasing a need for a new, more personal type of support^11^.

Peer-to-peer support programs offer individualized psychological and emotional support. Peer-to-peer professional programs connect an individual with a ‘peer’ who may have shared experiences of mental health or substance use, and have success in entering and maintaining their pathway to recovery^12^. Certified peer recovery coaches and peer support specialists have been trained to support others with similar experiences. Peer perceptions are vital to understanding the current landscape of tobacco cessation services.

Michigan is home to both rural and urban areas. Counties located in southeast Michigan are largely urban, while counties in northern areas are largely rural^13^. Tobacco use in rural areas is more prevalent compared to urban areas^14,15^. Peers residing in these areas may differ in their perspectives of common cessation supports due to differing tobacco use patterns in rural areas.

Since peers belong to a community with the lived experiences of mental/substance use diagnoses, they can share personal narratives of how their community is affected. Peers’ perceptions may be beneficial to inform training and strengthen support for tobacco cessation methods. The primary goal of this study is to investigate peers’ perceptions on currently available tobacco cessation supports and services, including any additional supports that may be necessary. This study also explores the perceptions of peers residing in rural areas compared to urban communities to identify specific challenges in tobacco cessation and to create opportunities to improve current and future cessation services.

## METHODS

### Participants

To assess peers’ needs related to smoking cessation, smoking risks, treatment options, and motives for treatment, a needs assessment was emailed to a specialty *listserv* in the state of Michigan. The survey was available from 30 November 2023 to 1 February 2024. Members of the *listserv* must be Michigan Department of Health and Human Services (MDHHS) certified as: 1) MDHHS-certified peer support specialists, 2) MDHHS-certified peer recovery coaches, and/or 3) Michigan Community Health Worker Alliance (MICHWA) community health workers. To maintain active certification, peers must complete continuing education of 32 hours every two years. As of the needs assessment survey distribution date, there were 2350 certified peer support specialists (i.e. mental health recovery primarily) and 1199 certified peer recovery coaches (i.e. substance use disorder recovery primarily and other co-occurring substance use disorders) on the *listserv*. This project was reviewed and deemed exempt from ethical approval by the University of Michigan Institutional Review Board.

### Tobacco cessation needs assessment

The peer needs assessment consisted of categorical items which were coded quantitatively. Self-reported data collected peers’ age range, county of residence, type of peer certification, smoking status (i.e. current, former, never user of tobacco products), smoking products used, cessation status (i.e. quit successfully, tried to quit, never quit), and respective perceptions of consequences of tobacco use. Perceptions of the health of other individuals who smoke were also assessed. The needs assessment asked respondents to rank the effectiveness of smoking supports (i.e. NRT, tobacco cessation counseling, Quitline services, medications, and peer-to-peer support). Respondents were also asked to indicate additional supports that may be valuable to increase smoking cessation rates. Further, the needs assessment collected perceptions on individuals who use multiple substances in addition to tobacco and on individuals taking medications for mental health conditions. The survey initially asked about peers’ gender identity, but this item was removed after respondent feedback to increase acceptability.

### Urban-rural locality

County data from the needs assessment were collected to assess urban or rural status, differences in perceptions among the various regions, and opportunities to increase appeal of tobacco cessation services. Urban counties were defined as counties with cities of greater than 50000 people, a county of at least 100000 people, or cities containing substantial community interchange of greater than 50000 people^13^. Counties were categorized rural if they did not meet the criteria for urban status.

### Statistical analysis

All measures of tobacco use history, peers’ perceptions on tobacco cessation, and tobacco cessation support were described via simple frequencies using SAS ver. 9.4^16^. Rurality status (e.g. rural vs urban) was cross-sectionally described by current and former smoking status and peer perceptions of different types of smoking supports. Continuous variables, such as age, were summarized. Missing values were excluded from analyses. A descriptive analysis was conducted and analyzed by rurality status to assess differences between rural and urban regions in Michigan. Findings are depicted in bar graphs.

## RESULTS

### Descriptive measures

The needs assessment was emailed to 2811 contacts, and received by 2717 individuals. Of the 2717 individuals, 501 opened the survey and 176 responded. Of the 176 responses, 4 survey submissions were considered as incomplete (i.e. answered less than two questions), resulting in a final sample of 172 responses. Of this sample, 137 peers reported holding one type of certification, 26 reported two types, and 6 reported three types. There were 105 MDHHS support specialists, 85 MDHHS recovery coaches, and 17 MICHWA community health workers. The age of respondents was as follows: 2 respondents (1.2%) were aged 18–24 years, 16 respondents (9.3%) were 25–34 years, 39 respondents (22.7%) were 35–44 years, 46 respondents (26.7%) were 45–54 years, 47 respondents (27.3%) were 55–64 years, and 22 respondents (12.8%) were ≥65 years.

We discovered that 53 peers (30.8%) currently use tobacco, 82 (47.7%) previously used tobacco, and 36 (21.5%) had no history of tobacco use. In total, 128 peers reported trying to quit tobacco previously; of these, 46 currently use tobacco (36.0%). Additionally, 7 peers (4.1%) who currently use tobacco have not tried to quit.

Among peers with past or current tobacco use, 35 (20.4%) previously used cigarettes, 27 (15.7%) use/used to use e-cigarettes or vapes, 3 (1.7%) use/used to use cigars, and 0 (0.0%) use/used to use chewing tobacco. When asked whether respondents had experienced negative consequences of tobacco use, 70 (51.9%) peers reported experiencing negative health consequences, while 41 (30.4%) did not, and 24 (17.8%) were unsure.

### Peers’ perceptions

When asked whether smoking was harmful to one’s health, 167 (97.7%) peers strongly agreed/agreed that smoking is harmful, while 3 (1.8%) neither agreed nor disagreed, and 1 (0.6%) strongly disagreed.

**Figure 1:**
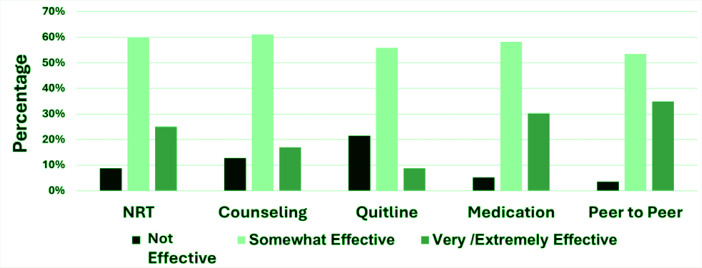
Ratings for smoking cessation supports

Peers rated several support services (i.e. Quitline, counseling, NRT, medication, and peer to peer) on their effectiveness to help people quit tobacco (Figure 1). The Quitline services were deemed not effective at all by 37 respondents (25.0%), while 96 (64.9%) thought it was somewhat effective, and 15 (10.1%) thought it was very/extremely effective. Tobacco cessation counseling was reported as not effective at all by 22 respondents (14.1%), somewhat effective by 105 respondents (67.3%), and very/extremely effective by 29 respondents (15.6%). Many respondents thought NRT was somewhat effective (n=103; 64.0%), while 15 (9.3%) did not think NRT was effective at all, and 43 (26.7%) thought it was very/extremely effective. Much like NRT, most respondents thought medications for smoking cessation were somewhat effective (n=100; 62.1%), 9 (5.6%) did not think medications were effective at all, and 52 (32.3%) thought they were very/extremely effective. Peer-to-peer support was rated as not effective by 6 respondents (3.8%), while majority of respondents thought it was somewhat effective (n=92; 58.2%) and 60 respondents thought it was very/extremely effective (38.0%).

**Figure 2:**
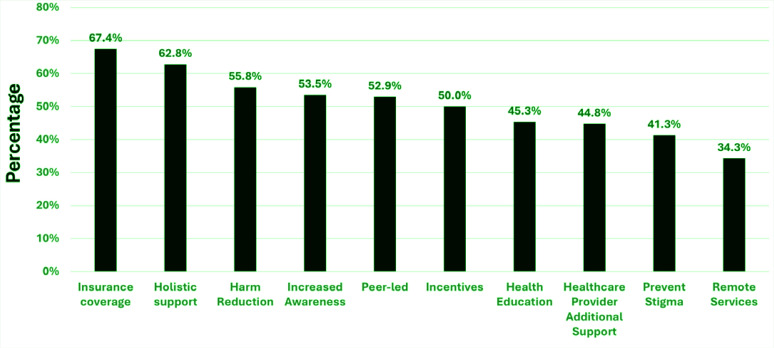
Ratings for additional smoking cessation supports

Peers rated additional support services on effectiveness to help people quit (Figure 2). A majority of peers advocate for more healthcare plans to pay for cessation services (n=116; 67.4%). Over half endorsed holistic support (i.e. total body wellness, yoga, acupuncture, nutrition; n=108; 62.8%), less focus on quitting and increased focus on harm reduction (n=96; 55.8%), integrated health (n=91; 52.9%), and/or peer-led groups (n=91; 52.9%). Half of respondents endorsed incentives, such as gift cards, should be provided to help people quit smoking (n=86; 50.0%). Fewer than half of peers supported more education about health impacts and negative consequences (n=78; 45.3%), additional support from healthcare providers (n=77; 44.8%), increased education from healthcare providers to prevent stigma towards people who use tobacco products (n=71; 41.3%), and remote/virtual opportunities such as telehealth and phone apps (n=59; 34.3%).

Peers rated how strongly they agreed or disagreed with the following statement: ‘Many people who use other substances (i.e. alcohol, cannabis, opioids) also smoke cigarettes’. A vast majority of peers strongly agreed/agreed (n=136; 82.4%), while 24 (14.6%) neither agreed nor disagreed, 4 (2.4%) disagreed, and one (0.6%) strongly disagreed. When asked how strongly they agree or disagree with the statement: ‘Many people taking medicine for their mental health condition(s) also smoke cigarettes’, the majority endorsed agreement as well: 93 (56.7%) peers strongly agreed/agreed, 58 (35.4%) neither agreed nor disagreed, 12 (7.3%) disagreed, and one (0.6%) strongly disagreed.

### Peers’ perceptions in urban and rural areas

**Figure 3:**
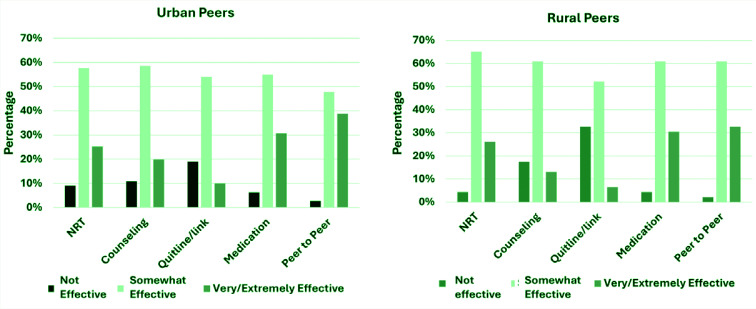
Urban vs rural peers perception on current supports

Among peers who currently use tobacco, 7 (4.1%) lived in rural areas and 46 (26.7%) lived in urban areas. More than half of urban-residing peers quit tobacco (n=81; 63.3%) compared to rural-residing peers (n=36; 28.1%). Rural-residing peers were more likely to find NRT somewhat effective (65.2%) compared to urban-residing peers (57.6%) (Figure 3). However, rural-residing and urban-residing peers were nearly equivalent in finding NRT very/extremely effective (rural: 26.1%; urban: 25.2%). Slightly more rural-residing peers viewed tobacco cessation counseling as somewhat effective (60.9%) than urban-residing peers (58.6%). Urban-residing peers were more likely to view counseling as very/extremely effective compared to rural-residing peers (urban: 19.8%; rural: 13.0%). Just over half of rural-residing and urban-residing peers were likely to view Quitline services as somewhat effective (rural: 52.2%; urban: 54.1%). More urban-residing peers thought the support was very/extremely effective compared to rural-residing peers (urban: 9.9%; rural: 6.5%). While rural-residing peers were more likely to view medications as somewhat effective (rural: 60.9%; urban: 55.0%), urban-residing and rural-residing peers similarly viewed them as very/extremely effective (urban: 30.6%; rural: 30.4%). Rural-residing peers were more likely to view peer-to-peer support as somewhat effective (60.9%). Urban-residing peers were more likely to view peer-to-peer support as very/extremely effective compared to rural-residing peers (urban: 38.7%; rural: 32.6%). More rural-residing peers endorsed other services would be very/extremely effective than peers residing in urban areas (rural: 15.2%; urban: 7.2%).

**Figure 4:**
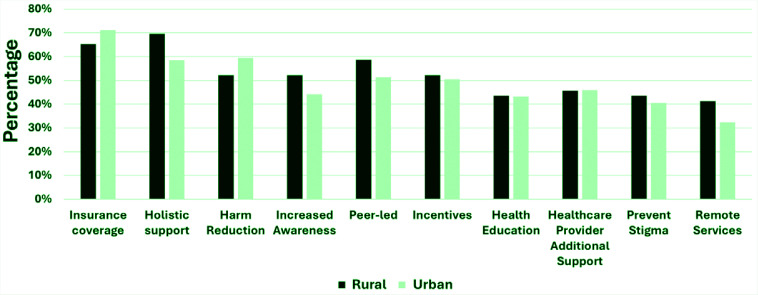
Additional supports needed to help more people successfully quit

We also evaluated peers’ perceptions of additional support options by rurality (Figure 4). Over half of all peers favored providing incentives to support tobacco cessation (rural: 52.2%; urban: 50.1%). Rural-residing peers were more likely to favor: 1) holistic support (rural: 69.6%; urban: 58.6%), 2) virtual/remote opportunities (rural: 41.3%; urban: 32.4%), and 3) peer-led groups (rural: 58.7%; urban: 51.4%). Urban-residing peers, however, were more likely to endorse less focus on quitting and more focus on any reduction in smoking compared to peers residing in rural areas (urban: 59.5%; rural: 52.2%). More rural-residing peers thought preventing stigma would be helpful compared to peers residing in urban areas (rural: 43.5%; urban: 40.5%). Both rural-residing and urban-residing peers similarly endorsed the need for more education for people who smoke cigarettes on how it impacts health and other negative consequences (rural: 43.5%; urban: 43.2%) and for the support of healthcare providers doing more to help people quit (rural: 45.7%; urban: 46%). Nearly half of all rural-residing peers endorsed more advertising/awareness about available cessation services is needed (rural: 52.2%; urban: 44.1%). More urban-residing peers favored healthcare plans paying for tobacco cessation services (urban: 71.2%; rural: 65.2%).

## DISCUSSION

This study provides needed information about peers and their perceptions of established and, additional supports for tobacco cessation. Additionally, peers’ perceptions of these supports were stratified by rurality in order to: 1) observe perceptions on tobacco cessation supports in both rural and urban areas in Michigan, and 2) identify opportunities for implementation of future supports for bolstering tobacco cessation among peers and ultimately those that they serve.

Most of the peers surveyed currently or previously used tobacco. Their lived experience of tobacco use and tobacco recovery can serve as an important function in implementing cessation services that will appeal to people who continue to smoke but seek recovery. A substantial portion of peers who currently use tobacco have attempted to quit, reflecting a desire of peers to reduce or quit tobacco.

Over half of all peer respondents perceived widely available tobacco cessation services (quitline, counseling, NRT, medications, peer-to-peer) as somewhat effective. Peer-to-peer support was perceived to be the most effective option to promote smoking cessation, suggesting that peers feel that a personal support system from others in recovery can be essential for successful tobacco cessation. In terms of additional tobacco cessation support, more than half of peers surveyed tended to favor financial accessibility, holistic health approaches, flexible approaches focused on harm reduction, increased awareness of available services, and peer support over other supports like health education, incentives, stigma prevention, additional support from a healthcare provider, and remote services. Peers may recognize financial barriers as obstacles to tobacco cessation and advocate for increased healthcare coverage to make cessation services more accessible^17,18^. Peers who endorse holistic health may be advocating for supports that go beyond the traditional services in place. Holistic health approaches can include various aspects of physical, mental, and emotional wellness and aid an individual in quitting tobacco. Flexible approaches shift the focus from solely quitting tobacco to preventing harm through reductions in use, reflecting more inclusive support for individuals in various stages of cessation. In addition, increased awareness (of cessation resources, methods, or support, etc.) may play a role in harm reduction. Peers may be highlighting the need for more adaptable and inclusive cessation methods. Peers also endorsed peer support which emphasizes the importance of incorporating social support and shared experiences into the cessation process.

Rural-residing and urban-residing peers differed in perceptions of tobacco cessation by their residence. More peers residing in rural areas reported perceiving current tobacco cessation services as at least somewhat effective, calling for greater access to these services and the need for novel approaches specifically for individuals in rural communities. Rural-residing peers endorsed a need for more holistic support, awareness of cessation services, virtual opportunities for care, peer-led services, and stigma prevention of traditional healthcare providers than urban-residing counterparts. Integrating these supports can address limited access to conventional services, heightened costs of cessation services, and emphasize community-centered approaches. Modest differences were found in the proportion of rural and urban-residing peers’ support of incentives, education on health and other consequences of tobacco use, and healthcare providers doing more to help people quit. Similar support of incentives may indicate that it be an acceptable element of cessation services, regardless of residency. Education on the health impacts of tobacco and other negative consequences and the role of healthcare providers is also valued as important in both rural and urban settings; however, more urban-residing peers endorsed harm reduction and more healthcare plans that pay for services.

### Limitations

This study includes a few limitations. First, this exploratory needs-assessment and analysis aimed to characterize perceptions of peers in Michigan. Analyses should be considered as exploratory and hypothesis-generating, and perspectives may differ in other states. Second, the assessment completion rate of those who opened the survey was modest (35.1%). Third, peers were not required to respond to every item in the needs assessment, so each item had a unique sample. Lastly, a few questions were centered around smoking specifically (as opposed to other forms of tobacco use). This wording could have been interpreted by peers with current or former tobacco use to omit a response to the question if they did not solely smoke a tobacco product.

## CONCLUSIONS

Exploring peers’ perceptions of tobacco use, tobacco’s impact on health, and cessation support services may aid in understanding the intersections between current supports, additional supports needed or desired, and challenges faced in rural and urban communities. Findings from this study suggest that peers believe there is a greater need for financially accessible, holistic, flexible, and community-based approaches to tobacco cessation for peers and potentially also for those they serve. Moreover, rural peers may advocate for increased awareness, holistic support, and virtual opportunities to support current and future tobacco cessation efforts.

## Data Availability

The data supporting this research are available from the authors on reasonable request.
